# Emotional cues from expressive behavior of women and men with Parkinson’s disease

**DOI:** 10.1371/journal.pone.0199886

**Published:** 2018-07-02

**Authors:** Shu-Mei Wang, Linda Tickle-Degnen

**Affiliations:** 1 Department of Rehabilitation Sciences, The Hong Kong Polytechnic University, Hung Hom, Kowloon, Hong Kong; 2 Department of Occupational Therapy, School of Arts and Sciences, Tufts University, Medford, Massachusetts, United States of America; KU Leuven, BELGIUM

## Abstract

**Objective:**

Emotional experience of people with Parkinson’s disease is prone to being misunderstood by observers and even healthcare practitioners, which affects treatment effectiveness and makes clients suffer distress in their social lives. This study was designed to identify reliable emotional cues from expressive behavior in women and men with Parkinson’s disease.

**Method:**

Videotaped expressive behavior of 96 participants during an interview of discussing enjoyable events was rated using the Interpersonal Communication Rating Protocol. Indices from emotional measures were represented in three components. Correlational analyses between expressive behavior domains and emotional components were conducted for the total sample and by gender separately.

**Results:**

More gross motor expressivity and smiling/laughing indicated more positive affect in the total sample. Less conversational engagement indicated more negative affect in women. However, women with more negative affect and depression appeared to smile and laugh more.

**Conclusion:**

This study identified reliable cues from expressive behavior that could be used for assessment of emotional experience in people with Parkinson’s disease. For women, because smiling/laughing may convey two possible meanings, that is, more positive and more negative affect, this cue needs to be interpreted cautiously and be used for detecting the intensity, not the type, of emotional experience. Healthcare practitioners should be sensitive to valid cues to make an accurate evaluation of emotion in people with Parkinson’s disease.

## Introduction

Parkinson’s disease affects dopamine-generating neurons in the substantia nigra of the basal ganglia and causes movement disorders [1]. The resulting movement symptoms, including bradykinesia (slowness of movements), muscle rigidity, and tremor, hamper expressive behavior. Abnormalities of emotional expressive behavior have been extensively documented in people with Parkinson’s disease (PD) [2–7]. Expressive behavior refers to the acts or movements involving channels via the face, body, voice, and speech that communicate a person’s inner emotion, personality, or motives [8,9]. During interpersonal interaction, conversational partners perceive these nonverbal or verbal cues from the person’s expressive behavior and accordingly interpret the person’s internal state [10–13]. Reduced expressivity easily misleads lay observers and health practitioners into identifying people with PD as being sad, hostile, or lacking emotion entirely, and into forming a possibly incorrect negative impression of them [14–16]. Indeed, studies [15–18] have shown that health practitioners are prone to making erroneous judgments of inner attributes in people with PD, which poses a threat to practitioner-patient rapport and effective treatment. Qualitative studies have indicated that people with PD suffer distress because of being misunderstood and negatively evaluated in every day social life [19–21], which emphasizes the need to enable observers to accurately interpret these individuals’ behavior.

Despite impairment in expressive behavior, people with PD have the potential to effectively convey their emotional experience in certain contexts [6,22–24], such as while they discuss enjoyable or pleasant topics and thus are facially expressive. Emotion literature [25,26] consistently indicates a two-dimension affective structure, that is, positive affect and negative affect, which are generally independent of each other (i.e. orthogonal). High positive affect includes high energy, enthusiasm, and pleasurable engagement, whereas low positive affect is characterized by lethargy [26]. Negative affect includes a variety of aversive emotions, such as distress and guilt. Low negative affect is characterized by calmness and tranquility [26]. Research findings [22,23] have shown that individuals with PD generally show more facial movement during contexts designed to elicit positive affect (e.g., talking about pleasant or enjoyable topics) compared with contexts which elicit negative affect (e.g., talking about unpleasant or frustrating topics). Additionally, it has been reported [24,27] that negative affect, compared with a calm state, exacerbates facial bradykinesia in people with PD, similar to the flat affect shown in the faces of depressed individuals. However, little is known about the association between emotional experience and expressive behaviors in the face, body, voice, and speech in people with PD. Identifying valid cues of positive affect, negative affect and depression from expressive behavior will help health professionals increase their sensitivity to valid cues, use these cues to accurately understand emotional experience in people with PD, and effectively adjust treatment plans.

It is also noteworthy that gender differences exist in expressive behavior in healthy people [28–30], although there is very little relevant research on women and men with movement disorders. Women generally are more nonverbally expressive than men because gender norms in society expect women to be emotionally expressive and to smile during interpersonal interactions, and expect men to suppress their emotional expression [29,31]. We anticipate that women and men with PD would follow the pattern of healthy people and differ in expressive movement. Indeed, the few studies [32,33] that have examined gender differences in expressive behavior among people with PD showed that women are more facially expressive than men. Further examination of potential differences in emotional cues between men and women with the disease will clarify whether or not practitioners must modify their use of cues according to gender.

To sum up, the primary purpose of this study was to identify valid emotional cues by examining associations between expressive behavior in the face, body, voice, and speech, and emotional experiences, including positive and negative ones, in people with PD. A secondary purpose of the study was exploratory, to begin to identify hypothesized gender differences and address this gap in the PD literature. This study had four research hypotheses, the first three addressing our primary purpose and the fourth our secondary purpose. First, we anticipated that expressive behavior would be associated with emotional experience, that is, expressive behavior would be valid cues of emotional experience in people with PD while they discussed enjoyable topics. Second, a higher degree of expressive behavior would indicate more positive emotional experience. Third, a lower degree of expressive behavior would indicate more negative emotional experience or more severe depression. We reasoned that individuals, even when discussing enjoyable topics, would vary in the degree to which they felt negative affect or were depressed. Fourth, we hypothesized that expressive behavior would be a stronger indicator of emotional experience in women than in men with PD. Specifically, the association between expressive behavior and emotional experience would be of larger magnitude in women than in men. This final hypothesis was exploratory in nature.

## Methods

### Research design

A correlational study design was used. We conducted the study by analyzing the baseline database from the American research study on *Self-Management Rehabilitation for PD* [22,34], which contains data on emotional experience, videotaped expressive behavior during an interview, demographic characteristics, and movement symptom severity (e.g., the motor examination section score of the Unified Parkinson’s Disease Rating Scale) (S1 Dataset).

### Participants

The participants in the current study met the following inclusion criteria, which came from the original research study: (1) a diagnosis of idiopathic PD confirmed by a neurologist; (2) ≥ 40 years old; (3) a score > 26 on the Mini-Mental Status Examination [35]; (4) Hoehn and Yahr stage 2.0–3.0 in “on” periods of responses to medications; (5) a score ≤ 20 on the Geriatric Depression Scale (GDS) [36]; (6) receiving stable doses of anti-Parkinson drugs for at least the 2 weeks immediately before the study began; (7) no difficulty in communicating with research personnel, assessed through direct observation of participants; and (8) no other severe diseases that might affect the participant’s movements. In order to be included in the current study, participants were those in the original research study who (1) had given written informed consent (on forms approved by the institutional review boards of Boston Medical Center and Boston University) to agree to the use of their data in studies other than the original research study; and (2) had no missing data concerning expressive behavior and emotional experience in the database.

### Procedure

At baseline, participants who passed screening completed a 30-minute videotaped interview protocol that involved self-reports about quality of life and enjoyable events in the past week in response to open-ended questions. Immediately after the interview, participants completed a self-report measure of their experienced affect during the interview.

### Measures of emotional experience

We included all measures from the database that addressed emotional experience. First, the Positive and Negative Affect Schedule (PANAS) [26] evaluated self-reported emotional experience during the interview. The PANAS includes 10 positive and 10 negative affect descriptors that require respondents to rate the extent of their experience of each specific affect descriptor on a five-point scale. A higher score reflects higher level of affective activation. Second, the GDS [36] evaluated depressive symptoms over the past week. The GDS is a self-report instrument and has 30 yes/no questions, with a higher total score being given for more severe depression. Due to the inclusion criterion (i.e., a GDS score ≤ 20) of the original database, the participants in the present study varied within the range of non-severe depression. Third, the Emotional Well-being subscale of the Parkinson’s Disease Questionnaire-39 items (PDQ-39) [37] measured self-reported emotional experience over the past month. The items of this subscale address attributes primarily related to depression and anxiety [38]. A higher score indicates more negative emotional experience. After principal component analysis (see S1 Table), the indices from all three emotional measures were divided into three emotional components: Positive Affect (PA; all positive affect indices of PANAS), Negative Affect (NA; all negative affect indices of PANAS), and Depression (all of the other indices). Three composite scores were created by averaging the participants’ z-scored responses to the indices comprising each component.

### Measures of expressive behavior

The Interpersonal Communication Rating Protocol (ICRP) [39] was used to rate facial, bodily, vocal, and verbal expressive behavior in people with PD. Several studies [18,22,40] have validated the use of ICRP items to evaluate expressive behavior in people with PD. The ICRP involved three measurement steps. First, a video camera filmed the face and upper body of people with PD, who were seated, during interviews. Second, short clips were extracted from the last 20 seconds of the interview videotapes when the participant described recent enjoyable events. The third step involved four trained raters separately rating each participant’s behavior during the clip. Participants’ clips were placed in random order on a DVD for viewing. Raters viewed the video channels of clips to rate facial and bodily behaviors, listened to the audio channels to rate vocal and two verbal behaviors (positive content and negative content), and used combined video and audio channels to rate the third verbal behavior “topic control”. Raters used a 5-point Likert scale (score 1 = low and 5 = high) to score their gestalt impression of the frequency, duration, and intensity of each ICRP item. Subsequently, the raters’ scores for each participant were averaged for each ICRP item. Effective reliability [41,42] was used to indicate how reliable the average score of four raters’ ratings was. The equation of effective reliability [41,42] is:

$$R_{SB}\;=\;\frac{nr}{1+(n-1)r}$$

where R_SB_ is *effective reliability* for the average score, *n* is the number of raters, and *r* is the average of the interrater reliability of all possible pairs of raters in a group of raters (called *mean reliability*). The average score from the four raters achieved adequate effective reliability on ICRP items (a mean of *R*_*SB*_ = 0.77) for the current study. Raters were blind to participants’ emotion scores to prevent rating biases. After principal component analysis (see S1 Supporting Information), all ICRP items were divided into six domains (Table 1) and integrated into one composite factor: Expressive Activation. Domain scores were created by averaging the scores of items that loaded on the same component. The Expressive Activation score was formed by averaging all ICRP scores after first reverse-scoring the forward slouching and tremors items, which were negatively correlated with the composite factor.

**Table 1:** Items of six expressive behavior domains.

Domain	ICRP item
Smile-Laugh	cheek raising, lip corner puller, laughing, and active facial expressivity
Conversational Engagement	gesturing with arms, vocal speed, active mouth closure during speech, talkativeness, and blinking
Vocal Acoustics	articulation, loudness, and vocal inflection
Gross Motor Expressivity	eyebrows raising, eyebrows pulling together, movements in trunk and head, and forward slouching
Confident Expressivity	tremors (reversely scored) and topic control (confident control of conversation)
Positivity of Speech Content	negative content (reversely scored) and positive content

*Note*. ICRP = the Interpersonal Communication Rating Protocol.

### Data analysis

Pearson’s correlation coefficients were calculated to examine the relationships between expressive behavior scores and emotional experience scores for the total sample, as well as for women, and men separately. Expressive behavior scores included Expressive Activation and the six expressive behavior domains. Emotional experience scores included Positive Affect, Negative Affect, and Depression. The *Z* test [43] was used to compare correlation coefficients between women and men. The alpha level of the statistical significance tests (two-tailed) for the correlational analysis and the *Z* test was set at 5% for statistical significance, and between 5% and 10% for trends toward statistical significance. We referred to Cohen’s criteria [44] for interpreting the magnitude of correlations, with the absolute value of 0.10 being considered “small”, 0.30 being considered “moderate”, and 0.50 being considered “large”.

## Results

### Participant characteristics

Among the original 116 participants in the original *Self-Management Rehabilitation for PD* database, 96 people (Table 2) met the inclusion criteria and were used in correlational analysis. The ratio of women (*n* = 26) to men (*n* = 70) in this study was 1:2.7, which is similar to the ratio (approximately 1: 2) of the PD population [45]. Men had more severe movement symptoms than women.

**Table 2:** Demographic and clinical data for the total sample (*N* = 96) and separately for women (*n* = 26) and men (*n* = 70).

Variable	Total sample	Women	Men	Gender difference
	Mean (SD)^a^	Mean (SD)^a^	Mean (SD)^a^	*t* or *χ*^*2*^
Age (year)	66.46 (9.07)	65.54 (9.09)	66.80 (9.10)	0.60
Time since diagnosis (year)	6.97 (5.36)^b^	6.96 (6.45)	6.97 (4.95)^b^	0.01
Caucasian, n (% within sample/gender)	94 (97.92%)	25 (96.15%)	69 (98.57%)	3.08
Mini-Mental Status Exam	29.30 (0.93)	29.58 (0.81)	29.20 (0.96)	-1.78
Hoehn & Yahr stage, n (% within sample/gender)				1.28
2.0	47 (48.96%)	15 (57.69%)	32 (45.71%)	
2.5	34 (35.42%)	7 (26.92%)	27 (38.57%)	
3.0	15 (15.63%)	4 (15.38%)	11 (15.71%)	
Unified Parkinson’s Disease Rating Scale				
Motor examination section^c^	23.03 (9.41)	18.65 (7.91)	24.66 (9.45)	-2.88**

^a^Unless otherwise indicated;

^b^One missing record in the dataset;

^c^A higher score indicates more severe motor impairment.

**p < 0.01.

### Expressive behavior correlates of Positive Affect

**Fig 1:**
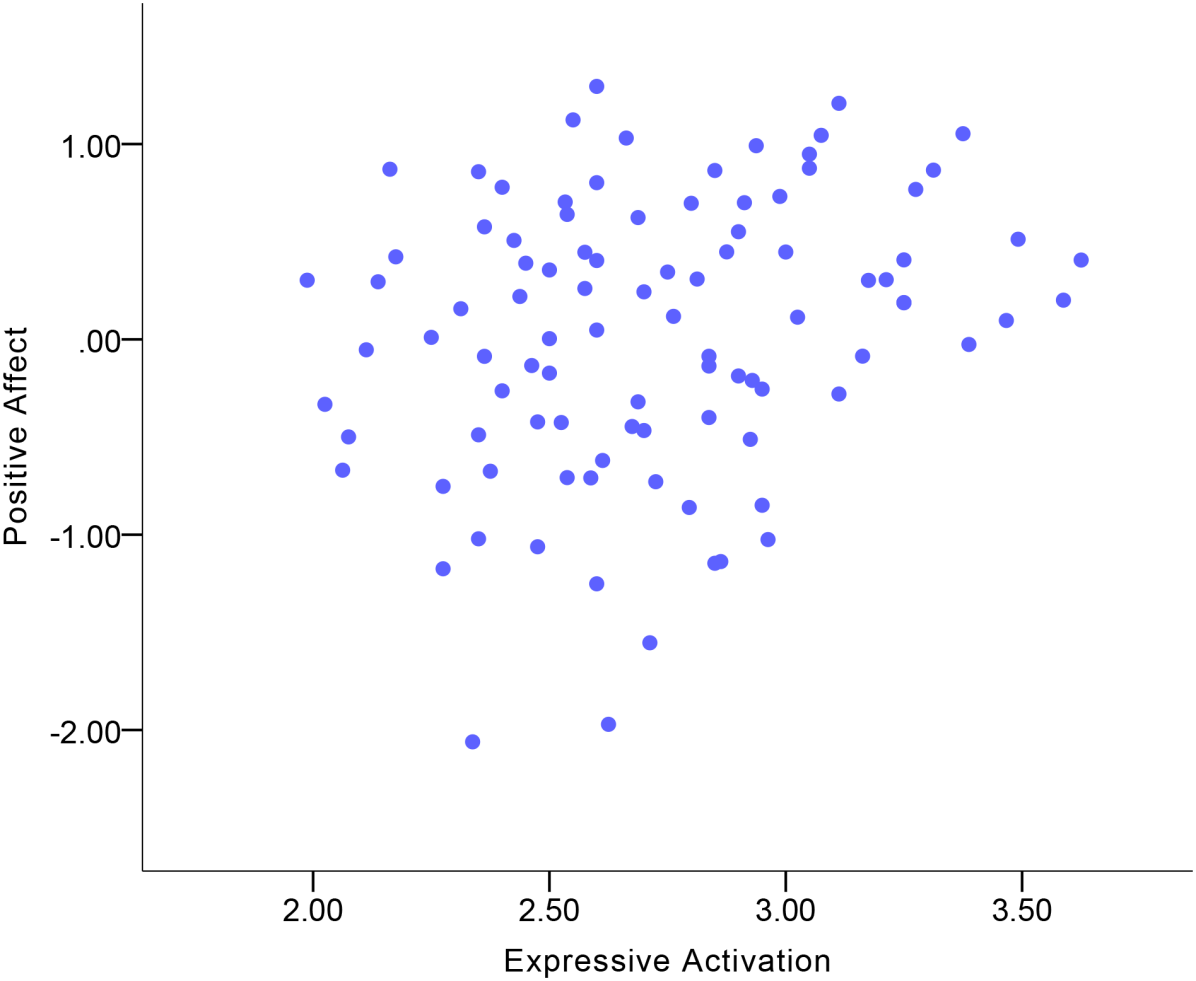
Correlation between Positive Affect and Expressive Activation in the total sample.

**Fig 2:**
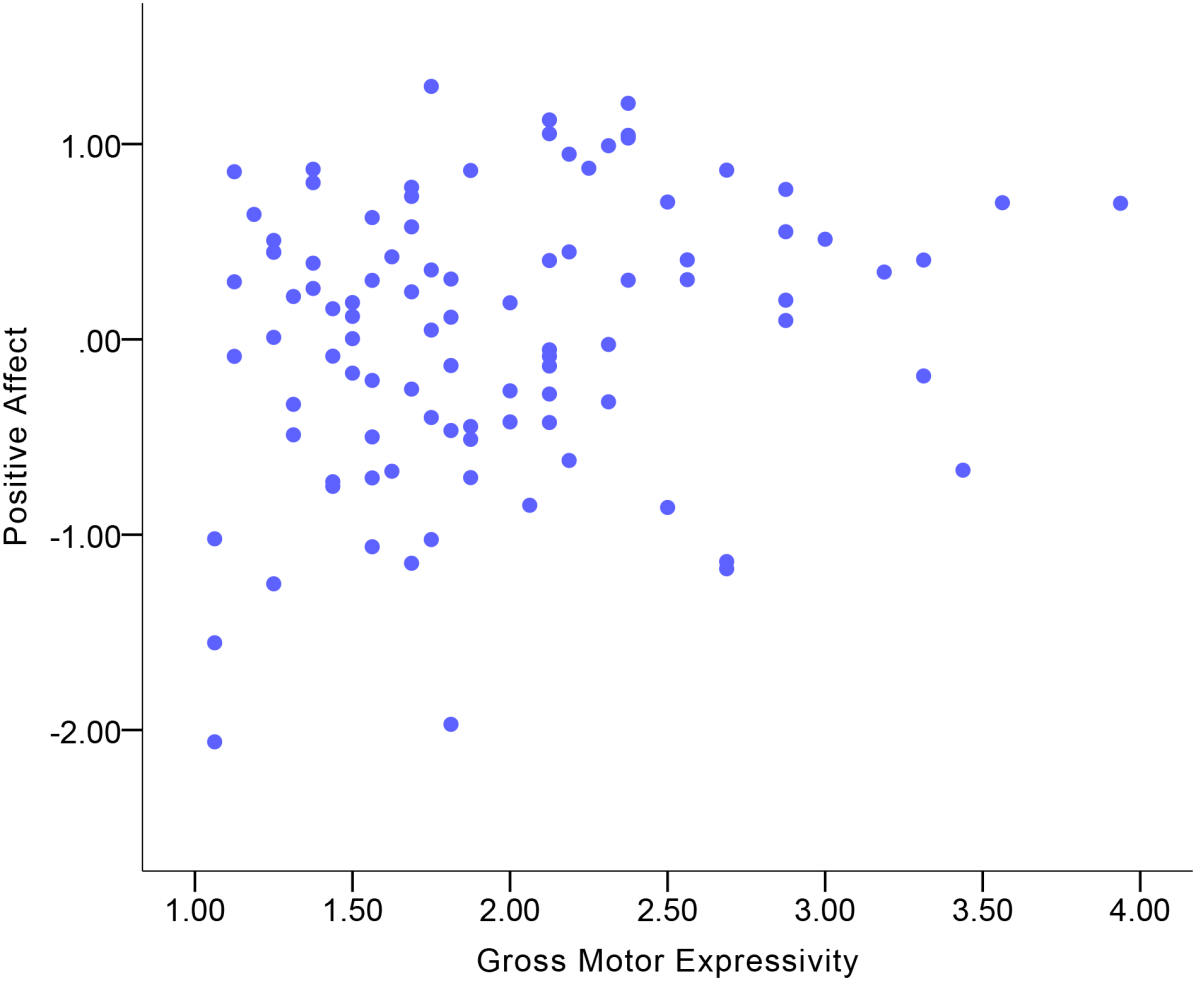
Correlation between Positive Affect and Gross Motor Expressivity in the total sample.

**Fig 3:**
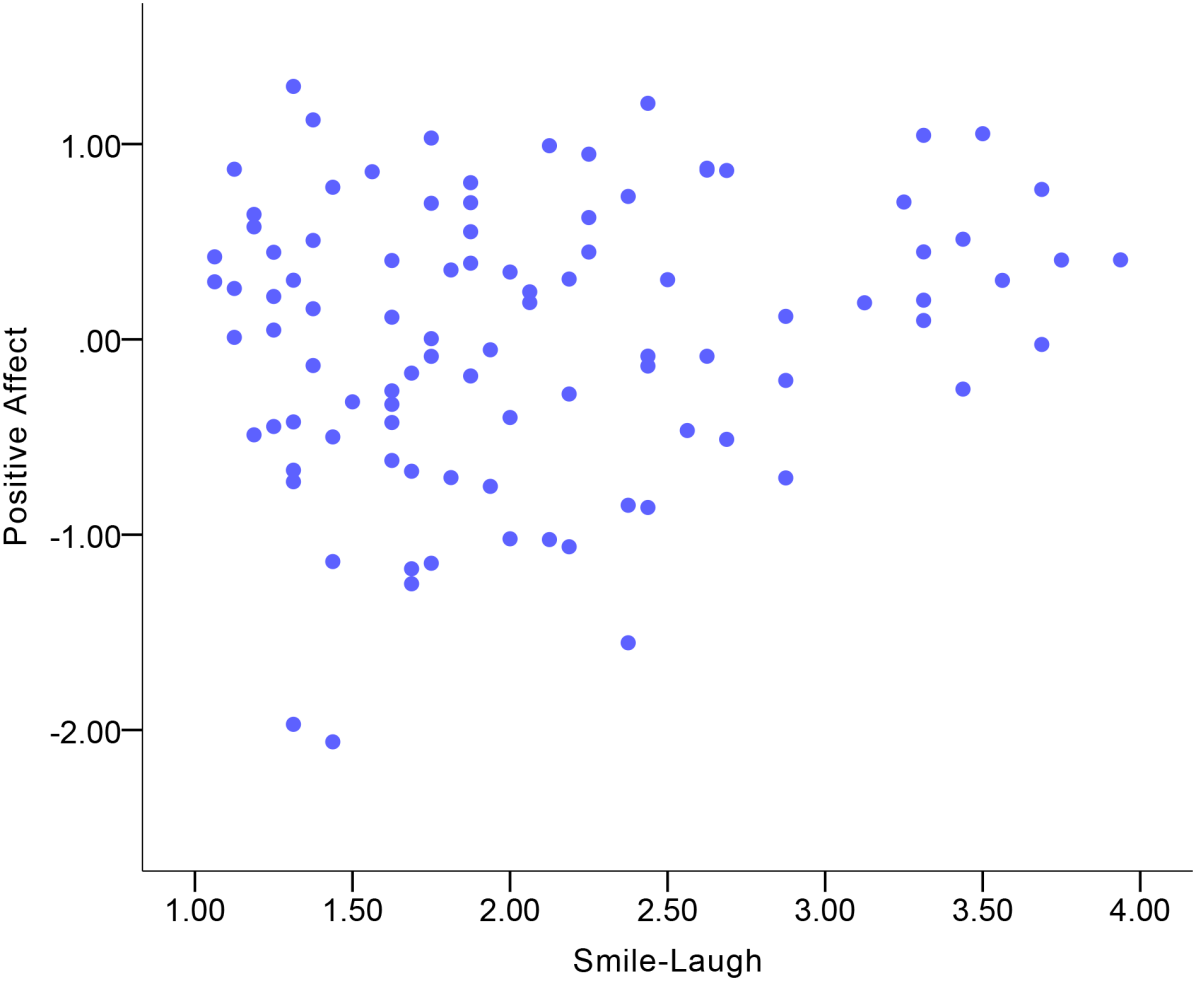
Correlation between Positive Affect and Smile-Laugh in the total sample.

Descriptive statistics of emotional experience and expressive behavior variables are displayed in S2 and S3 Tables. Table 3 shows the correlations between expressive behavior and Positive Affect. Expressive Activation was a small-to-moderate correlate of Positive Affect in the total sample (Fig 1). More Expressive Activation was indicative of more Positive Affect. Among the expressive behavior domains, Gross Motor Expressivity and Smile-Laugh were small-to-moderate correlates of Positive Affect in the total sample (Figs 2 and 3). More Gross Motor Expressivity and more Smile-Laugh were indicative of more Positive Affect.

**Table 3:** Correlation between expressive behavior and Positive Affect for the total sample (*N* = 96) and separately for women (*n* = 26) and men (*n* = 70).

Expressive behavior	Positive Affect			
	Sample			Gender difference
	Total sample	Women	Men	
	*r*	*r*	*r*	*Z*
Expressive Activation	0.24*	0.38^+^	0.20	0.84
Smile-Laugh	0.18^+^	0.34^+^	0.12	0.95
Conversational Engagement	0.12	0.20	0.10	0.42
Vocal Acoustics	0.16	0.14	0.19	-0.18
Gross Motor Expressivity	0.21*	0.43*	0.14	1.33
Confident Expressivity	0.02	0.01	0.03	-0.06
Positivity of Speech Content	0.13	0.25	0.08	0.74

*Note*. No significant or trend-level gender differences were found in correlations between expressive behavior and Positive Affect.

^+^ *p* < 0.10.

*p < 0.05.

**Fig 4:**
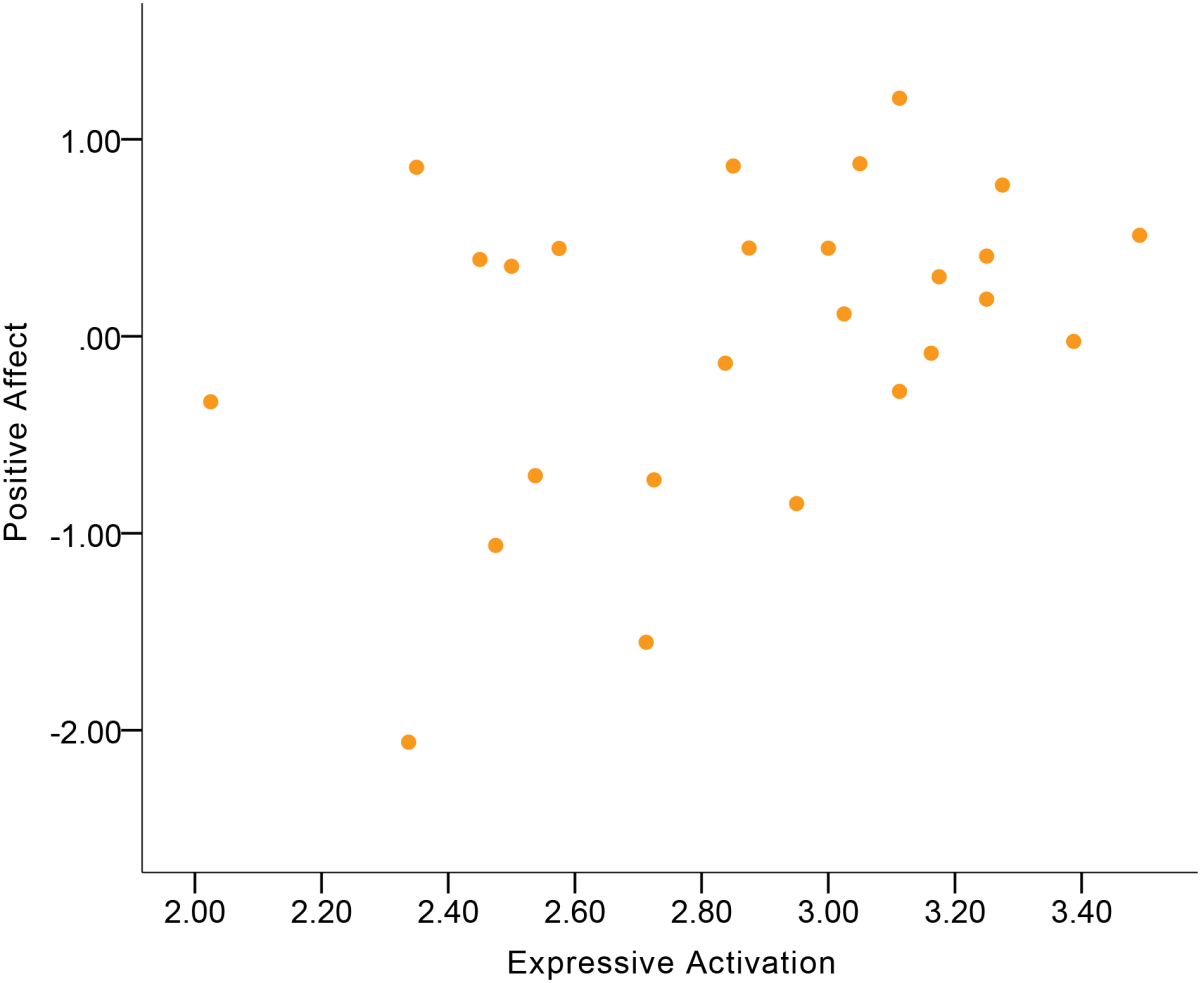
Correlation between Positive Affect and Expressive Activation in women.

**Fig 5:**
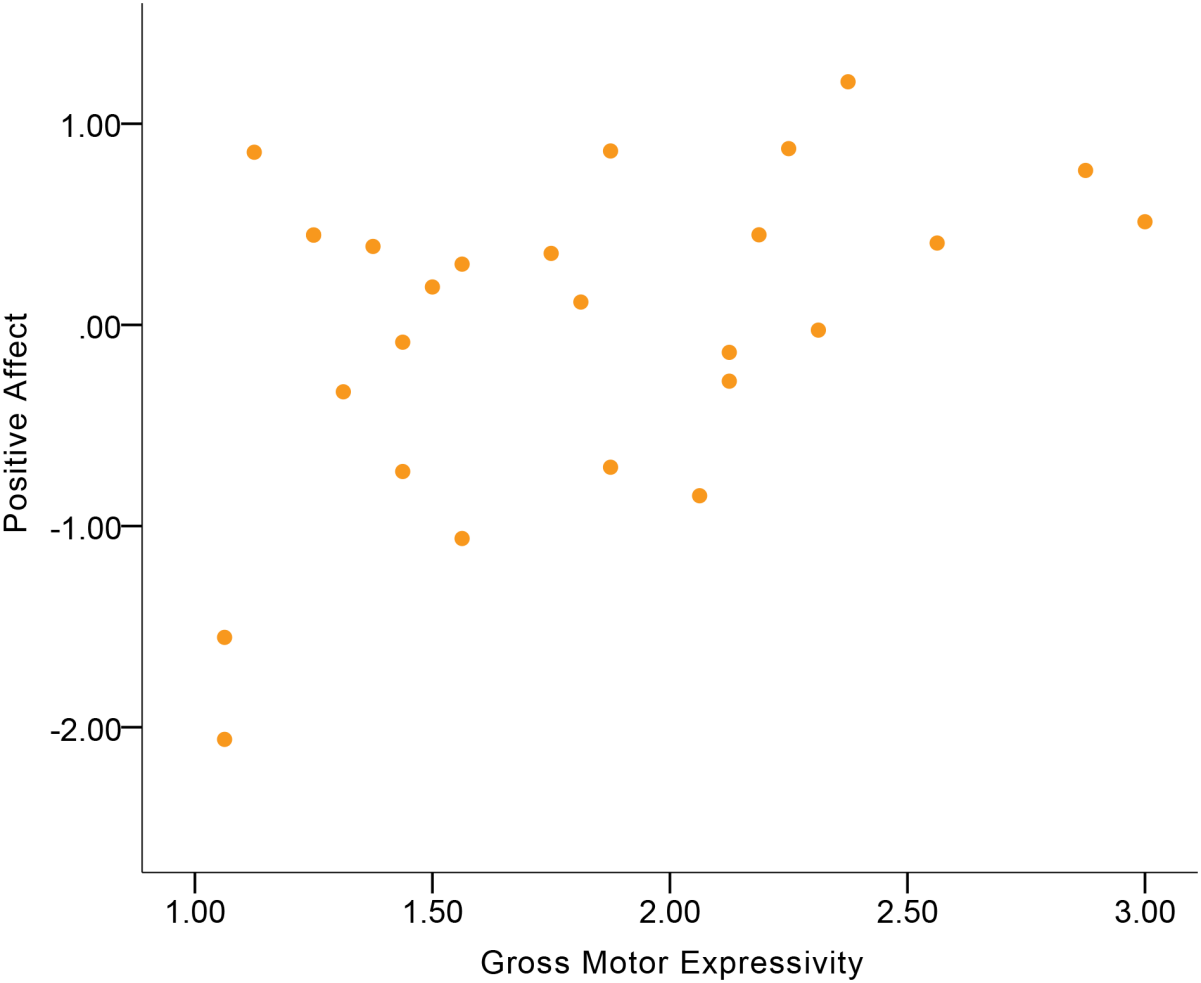
Correlation between Positive Affect and Gross Motor Expressivity in women.

**Fig 6:**
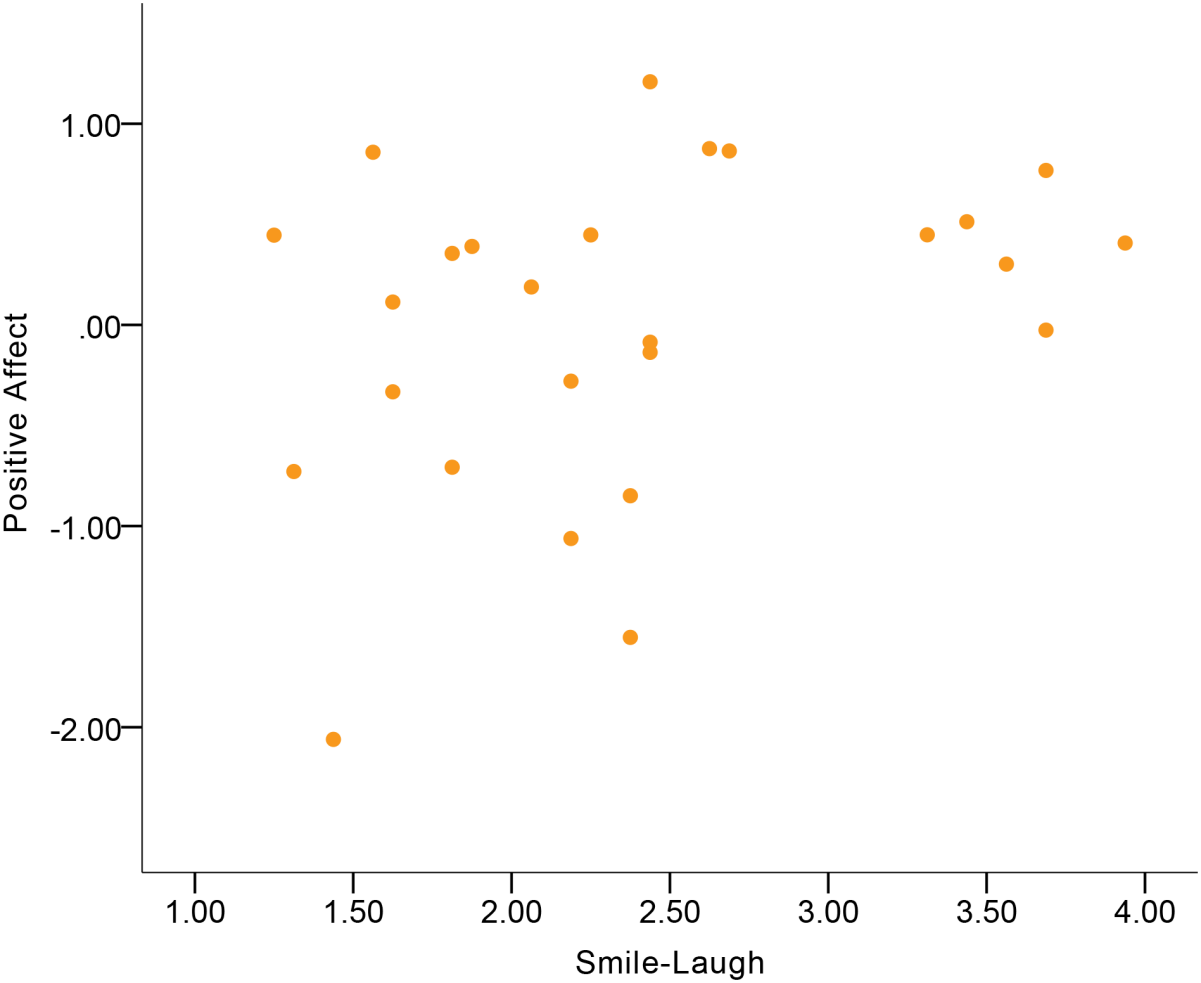
Correlation between Positive Affect and Smile-Laugh in women.

Although no significant gender differences were found in the correlations, the results showed that women’s Expressive Activation was indicative of Positive Affect at a moderate-to-large magnitude of positive correlation (Fig 4), while this correlation in men was at a small-to-moderate magnitude. For women, Gross Motor Expressivity and Smile-Laugh were moderate correlates of Positive Affect (Figs 5 and 6). More Gross Motor Expressivity and Smile-Laugh in women were indicative of more Positive Affect. For men, however, these two expressive behavior domains were small correlates of Positive Affect.

### Expressive behavior correlates of Negative Affect

**Fig 7:**
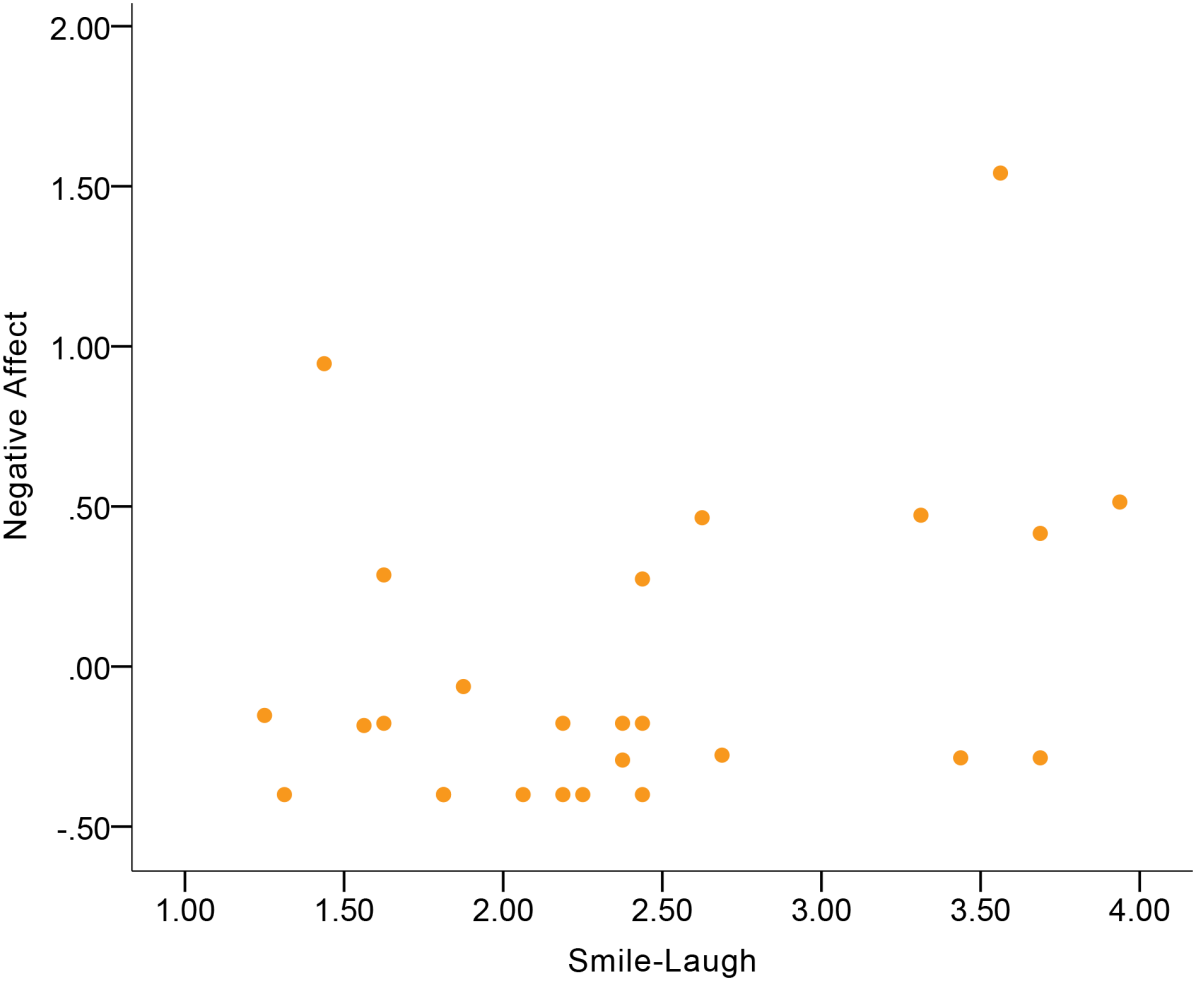
Correlation between Negative Affect and Smile-Laugh in women.

**Fig 8:**
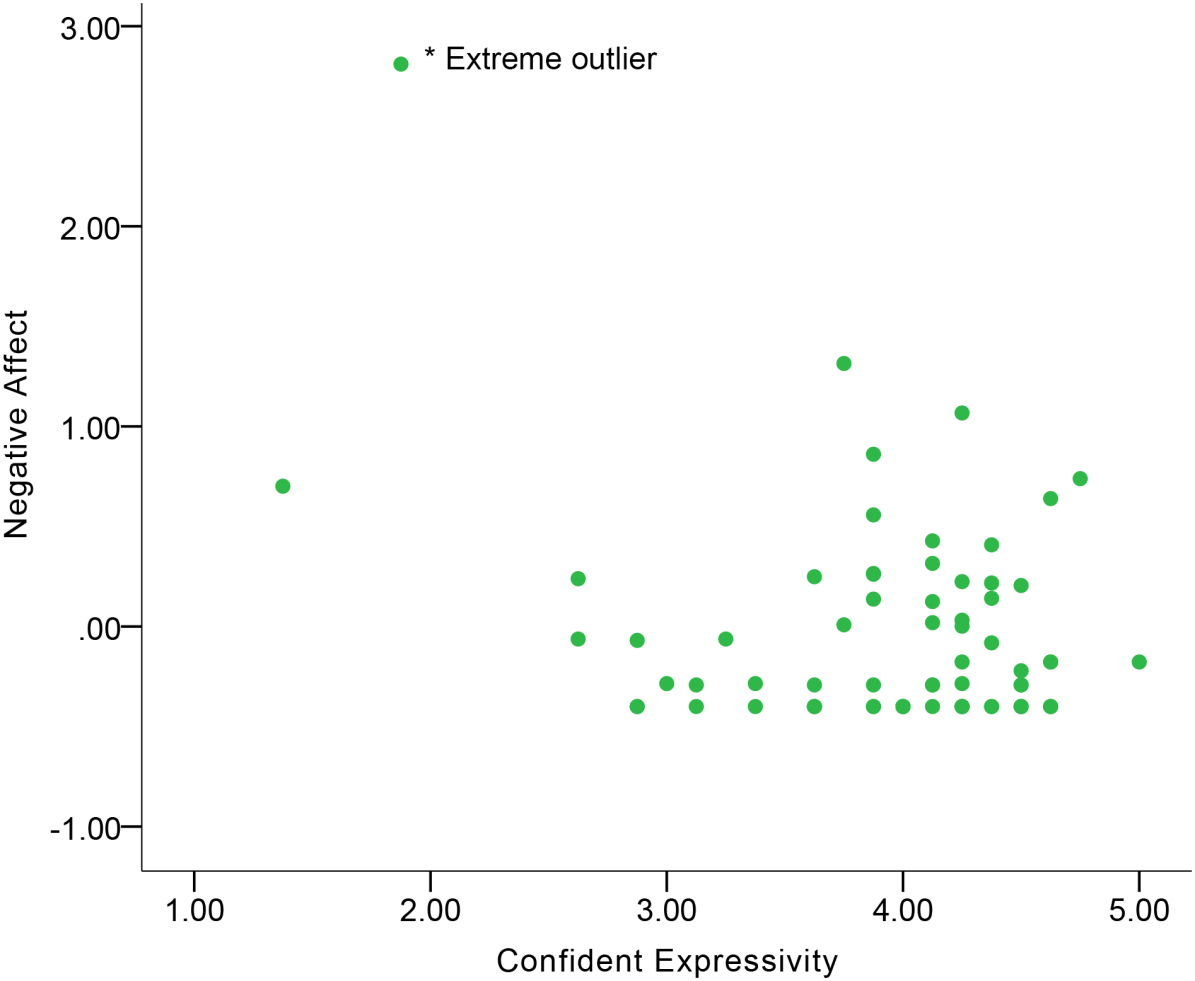
Correlation between Negative Affect and Confident Expressivity in men.

**Fig 9:**
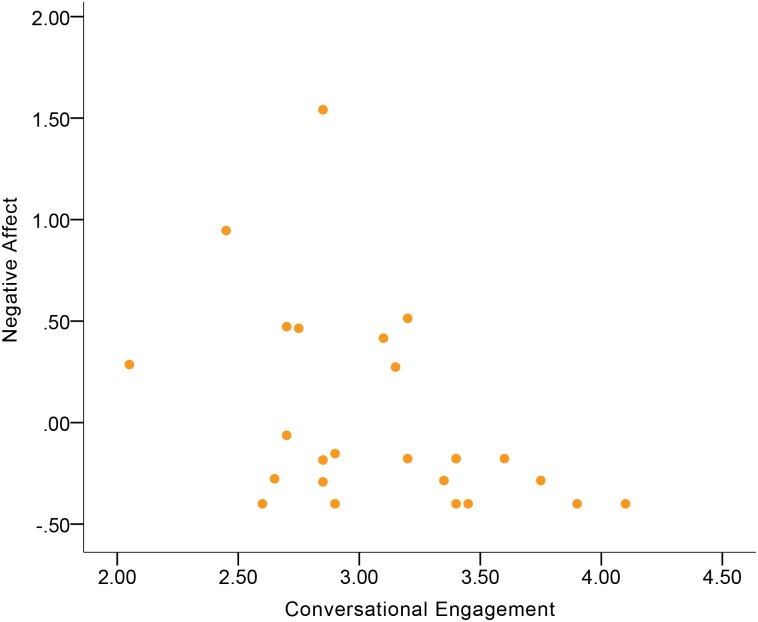
Correlation between Negative Affect and Conversational Engagement in women.

Table 4 shows the correlations between expressive behavior and Negative Affect. No significant correlations were found in the total sample. In terms of gender differences, women and men differed in the Smile Laugh-Negative Affect correlation and the Confident Expressivity-Negative Affect correlation. Women’s Smile-Laugh was indicative of Negative Affect at a moderate-to-large magnitude of positive correlation (Fig 7), but this correlation in men became negative at a small magnitude. Additionally, men’s Confident Expressivity was indicative of Negative Affect at a moderate magnitude of negative correlation (Fig 8), while this correlation in women became positive at a moderate magnitude. Although no significant gender difference was found in the Conversational Engagement-Negative Affect correlation, the results showed that women’s Conversational Engagement was indicative of Negative Affect at a moderate-to-large magnitude of negative correlation (Fig 9), while this correlation in men was at a small magnitude.

**Table 4:** Correlation of expressive behavior with Negative Affect and Depression for the total sample (*N* = 96) and separately for women (*n* = 26) and men (*n* = 70).

Expressive behavior	Negative Affect				Depression			
	Sample			Gender difference	Sample			Gender difference
	Total	Women	Men		Total	Women	Men	
	*r*	*r*	*r*	*Z*	*r*	*r*	*r*	*Z*
Expressive Activation	-0.05	0.04	-0.10	0.59	0.07	0.27	-0.01	1.18
Smile-Laugh	0.01	0.36^+^	-0.13	2.13*	0.13	0.36^+^	0.04	1.42
Conversational Engagement	-0.14	-0.42*	-0.06	-1.58	0.05	0.15	0.01	0.58
Vocal Acoustics	0.06	-0.08	0.09	-0.72	-0.12	<0.01	-0.19	0.83
Gross Motor Expressivity	0.08	-0.01	0.11	-0.48	0.13	0.20	0.13	0.32
Confident Expressivity	-0.17	0.28	-0.30*^a^	2.46*^b^	-0.01	<0.01	-0.02	0.10
Positivity of Speech Content	0.04	0.03	0.04	-0.01	0.13	0.13	0.12	0.03

^a^This finding is *r* = -0.07 (*p* = 0.56) when an outlier in men is removed.

^b^*Z* = 1.49 (*p* = 0.14) when an outlier in men is removed.

^+^ *p* < 0.10.

*p < 0.05.

Visual inspection of correlation figures in terms of Negative Affect (Figs 7 to 9) suggested the possibility of extreme outliers. In order to check if extreme outliers drove the aforementioned correlation results of Negative Affect in women and men, we conducted an extreme outlier analysis based on examination of variable boxplots in both genders in IBM SPSS Statistics 23. An extreme outlier, which was outside three times the distance of interquartile range of data [46], was found in Negative Affect of men. After we eliminated that extreme outlier participant and re-conducted the Pearson’s correlation, the Confident Expressivity-Negative Affect correlation in men became non-significant (*r* = -0.07, *p* = 0.56, *n* = 69). Consequently, the gender difference in the Confident Expressivity-Negative Affect correlations became non-significant (*Z* = 1.49, *p* = 0.14). In addition to eliminating the extreme outlier and re-running the Pearson’s correlation, another strategy to handle the extreme outlier is to conduct the Spearman’s correlation on the entire sample of men including the outlier (*n =* 70). The result was consistent with the finding when we eliminated the outlier by showing that the Confident Expressivity-Negative Affect correlation in men was non-significant (*r* = -.07, *p* = .58).

### Expressive behavior correlates of Depression

**Fig 10:**
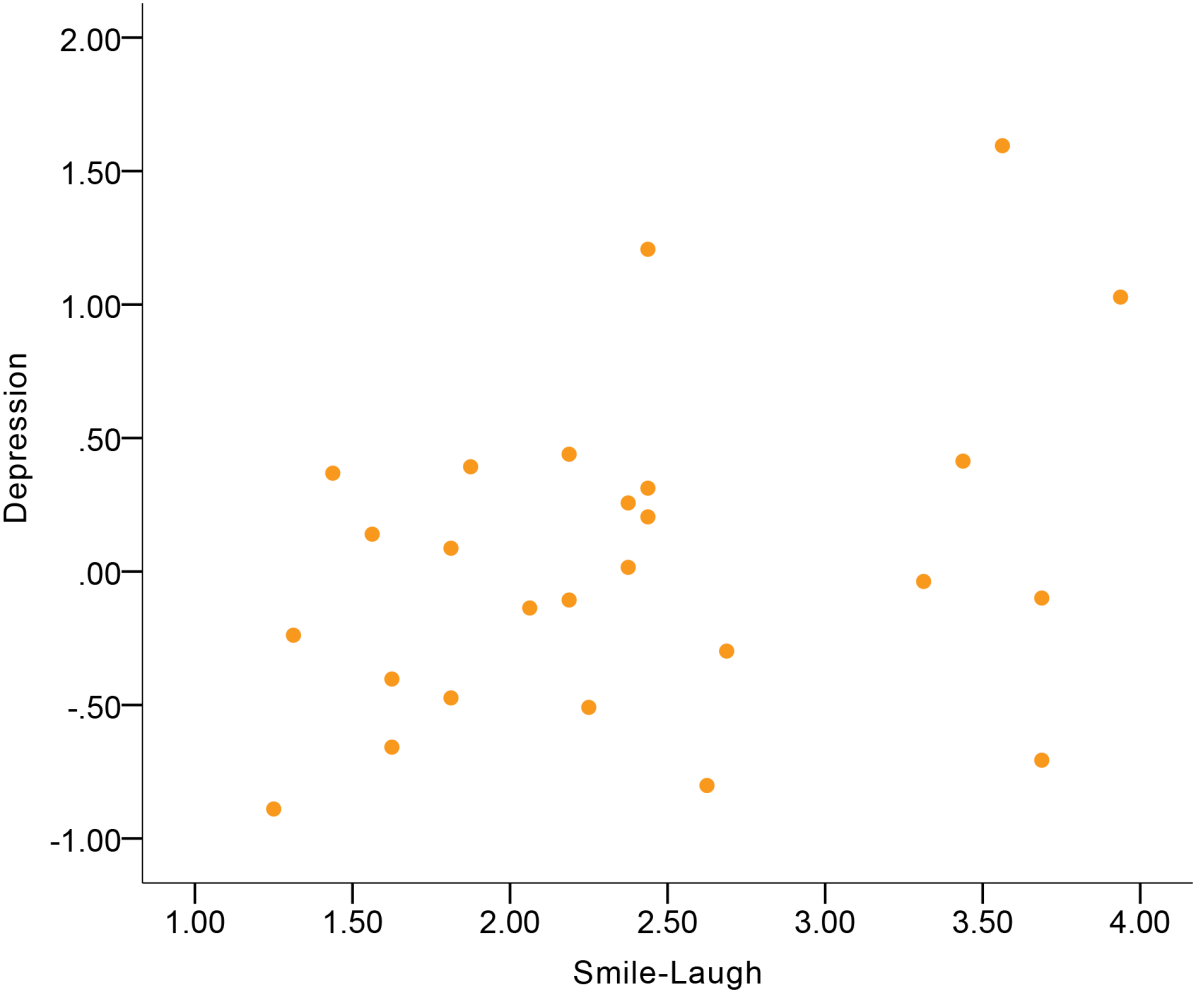
Correlation between Depression and Smile-Laugh in women.

No significant correlations were found in the total sample (Table 4). Although no significant gender differences were found, women’s Smile-Laugh was indicative of Depression at a moderate-to-large magnitude of positive correlation (Fig 10), while this correlation in men was near-zero.

## Discussion

The findings of this study, supporting the first hypothesis, indicate that valid cues of emotional experience exist in the expressive behavior of people with PD. The results are consistent with earlier literature [6,22–24] showing that even though expressive behavior is impaired, it continues to have the potential to effectively convey attributes in PD people. We extend earlier results by finding that expressive behavior served as valid emotional cues for this population. An increase in gross motor expressivity was indicative of more positive affect in people with PD. More conversational engagement was indicative of less negative affect in women. It is noteworthy that smiling and laughing appeared to be valid indicators of more positive affect as well as more negative affect and depression in women.

In line with our second hypothesis, a higher degree of expressive behavior indicated more positive affect in people with PD while they were discussing enjoyable topics, which is consistent with earlier work [22,23,47]. An increase in gross motor expressivity, smiling and laughing were reliably indicative of more positive affect in the PD population. Based on our findings and previous studies of personality cues [17,40] showing a linkage between brow furrowing and neuroticism, it is suggested that gross motor expressivity may serve as an important cue of inner attributes in people with PD. Additionally, smiling and laughing are plausible cues of positive affect because smiles are linked to enjoyment [48–50], while laughter (which co-occurs often with smiling) is a multifaceted behavior that can occur in many emotional situations including happiness, nervousness, or sadness [51–53].

The third research hypothesis was that a lower degree of expressive behavior indicated more negative affect and more severe depression in people with PD while they discussed enjoyable topics. However, our findings only partially support this hypothesis. The result of conversational engagement in women supports the hypothesis: less conversational engagement indicated more negative affect, which is in accord with earlier reports [54,55] indicating the linkage between fewer gestures and the lack of enthusiasm, as well as between slower speaking and sadness. The finding that fails to support the third hypothesis is that more smiling and laughing were also indicative of more negative affect and depression in addition to more positive affect in women, which may be explained by women’s interpersonal motivations. Literature [28,56] has suggested that women seem to be more concerned with social approval, affiliation, and making good impressions via smiling than men, thus making their smiles less reliable as an indicator of genuine enjoyment than those in men. In our study, social norms may have put pressure on people with PD to behave happily under the condition of discussing enjoyable events with an interviewer. Therefore, in order to conform to the social expectations and win social approval, women with more negative affect or depression may have disguised their negative emotional experience by demonstrating more smiles and laughter. This notion is supported by earlier studies [33,57,58] indicating that people with PD have the ability, although it is impaired compared with that in healthy people, to pose facial expression and mask negative emotion.

This fourth hypothesis was exploratory in nature. We hypothesized that expressive behavior was a stronger indicator of emotional experience in women than in men. Generally this pattern can be observed from the magnitudes of the correlational associations, which may be because women indeed are more nonverbally expressive than men [28–31,33]. Moreover, we found that the genders presented contrasting smiling and laughing behavior when experiencing more negative affect, suggesting that gender differences not only appear in expressive intensity, but also appear in ways of conveying negative emotional experience.

It is noteworthy that men had more severe movement symptoms than women in this study. A possibility deserving comment is that expressive behavior showed a pattern of being a stronger cue of emotional experience in women than in men perhaps due to the former’s milder movement symptoms and not due to the gender factor that we proposed in the third hypothesis. In order to exclude this explanation, we re-examined the correlations between expressive behavior and emotional experience by computing partial correlation coefficients controlling for movement symptom severity measured by the motor examination section of the Unified Parkinson’s Disease Rating Scale. These partial correlation analyses did not change the findings compared to the uncontrolled correlational analyses. Therefore, the evidence supports the third hypothesis.

### Limitations and future research

First, our sample was unable to include people with severe depression due to the inclusion criteria of the original database, which limited depression variation in the correlational analysis and thus the ability to detect emotional indicators from expressive behavior. Future research should include a heterogeneous sample with varying depression levels to identify possible indicators of the emotional experience. Second, difficulty in identifying feelings, which affects the self-report of emotional experience, may exist in people with PD [59–61]. However, this study analyzed the already-collected data from the research database on Self-Management Rehabilitation for people with Parkinson’s disease [22,34], which did not directly assess whether the emotion-identifying difficulty existed in the participants. Nevertheless, this study used the Mini-Mental Status Examination in the screening step to ensure that participants had generally intact cognitive status, which is related to ability to identify feelings and complete self-report of emotional experience. In addition, participants in this study were in the earlier stages of the disease and functioning generally independently in the community, further supporting their ability to report their feelings. Therefore, the emotional data in this study are valid. Future studies that directly assesse emotion-identifying deficits in PD people and screen out unsuitable individuals will provide more robust evidence to justify the use of self-reported emotional data. Furthermore, this study focused on identifying valid emotional cues in people with PD. The next step is to examine the degree to which health practitioners use valid cues to evaluate these clients’ emotional experience. This study provides an exploratory report of gender differences in emotional cues in people with PD. Future research may adopt a large sample size of both women and men with PD to conduct a high-powered hypothesis test of our findings.

### Implications

- Health practitioners should be sensitive to and use gross motor expressivity to accurately evaluate positive affect in people with PD.
- Practitioners should observe conversational engagement in women to accurately evaluate negative affect.
- Smiling and laughing may serve as valid cues to indicate women’s higher intensity of emotional experience, regardless of positive affect or negative affect, which is crucial to activating the subsequent detailed emotional assessment. Practitioners who note more smiling and laughing in women could further probe with specific questions about positive and negative affect and gather women’s verbal disclosure to assess their emotional experience in a detail way [62]. Additionally, practitioners could rely on the combination of smile-laugh and other valid cues, such as the aforementioned gross motor expressivity and conversational engagement, to distinguish emotional types in women. Specifically, more smiling and laughing along with more gross motor expressivity reflect more positive affect. More smiling and laughing along with less conversational engagement indicate more negative affect in women.
- Professional training of practitioners may involve sensitization to the valid emotional cues from expressive behavior in people with PD.
- Practitioners may educate family caregivers of clients to observe the valid emotional indicators when they communicate with clients with PD. This family education helps reduce clients’ social distress because they could feel that their emotional experience is understood correctly by important others.

## Conclusion

The major contribution of this study is that it identifies valid emotional cues from different domains of expressive behavior in people with PD while this population discussed enjoyable topics. The cues included (1) gross motor expressivity for positive affect in people with PD, and (2) conversational engagement for women’s negative affect. This study also suggests that smiling and laughing may be used to assess emotional intensity, but not different types of emotional experience, in women because more smiling and laughing in women may reflect more positive affect as well as more negative affect and depression. These results provide a basis for future studies examining whether practitioners indeed use these valid cues to accurately evaluate emotional experience in people with PD. Accurate evaluation presumably would contribute to quality improvement of treatment. Health practitioners may increase their sensitivity to using these valid cues to facilitate effective emotional evaluation and meaningful intervention for these clients.

## Supporting information

S1 Table Rotated component loadings for each emotional index in the three-component solution of the principal component analysis with varimax rotation for people with Parkinson’s disease (*N* = 105^a^). (DOCX)

S2 Table Descriptive statistics of emotional measures for the total sample (*N* = 96) and separately for women (*n* = 26) and men (*n* = 70). (DOCX)

S3 Table Descriptive statistics of expressive behavior variables for the total sample (*N* = 96) and separately for women (*n* = 26) and men (*n* = 70). (DOCX)

S1 Supporting Information Steps to develop expressive behavior domains. (DOCX)

S1 Dataset Dataset of this study. (SAV)
