# Supplementary material for: Emotional cues from expressive behavior of women and men with Parkinson’s disease
Source: PLoS One. 2018 Jul 2;13(7):e0199886. doi: 10.1371/journal.pone.0199886 (PMC6028092; doi:10.1371/journal.pone.0199886)
Supplement: S1 Table — (DOCX) [file pone.0199886.s001.docx]

**S1 Table.** **Rotated component loadings for each emotional index in the three-component solution of the principal component analysis with varimax rotation for people with Parkinson’s disease (*N* = 105^a^).**

| Emotional index | Emotional component | | |
| --- | --- | --- | --- |
|  | Positive Affect | Negative Affect | Depression |
| PANAS-PA: Enthusiastic | 0.78 |  |  |
| PANAS-PA: Strong | 0.77 |  |  |
| PANAS-PA: Excited | 0.76 |  |  |
| PANAS-PA: Active | 0.73 |  |  |
| PANAS-PA: Proud | 0.72 |  |  |
| PANAS-PA: Determined | 0.71 |  |  |
| PANAS-PA: Inspired | 0.69 |  |  |
| PANAS-PA: Interested | 0.65 |  |  |
| PANAS-PA: Alert | 0.61 |  |  |
| PANAS-PA: Attentive | 0.56 |  |  |
| PANAS-NA: Hostile |  | 0.78 |  |
| PANAS-NA: Ashamed |  | 0.75 |  |
| PANAS-NA: Upset |  | 0.75 |  |
| PANAS-NA: Irritable |  | 0.73 |  |
| PANAS-NA: Jittery |  | 0.52 |  |
| PANAS-NA: Distressed |  | 0.47 |  |
| PANAS-NA: Guilty |  | 0.40 |  |
| PANAS-NA: Scared |  | 0.34 |  |
| PANAS-NA: Afraid |  | 0.34 |  |
| PANAS-NA: Nervous |  | 0.34 |  |
| PDQ-39: Depressed |  |  | 0.81 |
| PDQ-39: Anxious |  |  | 0.78 |
| PDQ-39: Worried about the future |  |  | 0.69 |
| PDQ-39: Isolated and lonely |  |  | 0.69 |
| GDS |  |  | 0.69 |
| PDQ-39: Angry or bitter |  |  | 0.57 |
| PDQ-39: Weepy or tearful |  |  | 0.49 |
| Eigenvalue | 5.26 | 3.74 | 3.47 |
| Explained variance (%) | 19.49 | 13.85 | 12.84 |
| Cronbach’s alpha | 0.89 | 0.76 | 0.82 |

*Note.* PANAS-PA = the Positive and Negative Affect Schedule-Positive Affect; PANAS-NA = the Positive and Negative Affect Schedule-Negative Affect; GDS = the Geriatric Depression Scale; PDQ-39 = the Parkinson’s Disease Questionnaire-39 items. The items within the Emotional Well-being subscale of PDQ-39 are listed.

^a^In addition to 96 people, the extra nine people only having some missing data either in emotional indices or in expressive behavior items were also included in the principal component analysis in order to have a larger sample size.
