# Supplementary material for: Emotional cues from expressive behavior of women and men with Parkinson’s disease
Source: PLoS One. 2018 Jul 2;13(7):e0199886. doi: 10.1371/journal.pone.0199886 (PMC6028092; doi:10.1371/journal.pone.0199886)
Supplement: S2 Table — (DOCX) [file pone.0199886.s002.docx]

**S2 Table. Descriptive statistics of emotional measures for the total sample (*N* = 96) and separately for women (*n* = 26) and men (*n* = 70).**

| Emotional measure^a^ | Total sample | Women | Men |  | Gender difference |
| --- | --- | --- | --- | --- | --- |
|  | Mean (SD) | Mean (SD) | Mean (SD) |  | *t* |
| PANAS |  |  |  |  |  |
| Positive Affect | 36.03 (7.72) | 35.73 (8.43) | 36.14 (7.50) |  | -0.23 |
| Negative Affect | 12.68 (3.42) | 12.77 (3.28) | 12.64 (3.49) |  | 0.16 |
| GDS | 6.34 (4.22) | 6.85 (4.00) | 6.16 (4.31) |  | 0.71 |
| PDQ-39 |  |  |  |  |  |
| Emotional Well-being | 7.18 (3.99) | 7.62 (3.45) | 7.01 (4.18) |  | 0.66 |

*Note.* PANAS = the Positive and Negative Affect Schedule; GDS = the Geriatric Depression Scale; PDQ-39 = the Parkinson’s Disease Questionnaire-39 items. A higher score indicates a greater degree of positive affect and negative affect in PANAS, and more depression in GDS and the Emotional Well-being of the PDQ-39. No significant gender differences were found in emotional measures.

^a^The sum of items within each measure was calculated.
