# Supplementary material for: Emotional cues from expressive behavior of women and men with Parkinson’s disease
Source: PLoS One. 2018 Jul 2;13(7):e0199886. doi: 10.1371/journal.pone.0199886 (PMC6028092; doi:10.1371/journal.pone.0199886)
Supplement: S3 Table — (DOCX) [file pone.0199886.s003.docx]

**S3 Table. Descriptive statistics of expressive behavior variables for the total sample (*N* = 96) and separately for women (*n* = 26) and men (*n* = 70).**

| Expressive behavior | Total sample | Women | Men |  | Gender difference |
| --- | --- | --- | --- | --- | --- |
|  | Mean (SD) | Mean (SD) | Mean (SD) |  | *t* |
| Overall evaluation |  |  |  |  |  |
| Expressive Activation | 2.72 (0.37) | 2.87 (0.37) | 2.67 (0.36) |  | 2.40* |
| Domain |  |  |  |  |  |
| Smile-Laugh | 2.07 (0.75) | 2.38 (0.79) | 1.95 (0.70) |  | 2.60* |
| Conversational Engagement | 2.95 (0.47) | 3.08 (0.47) | 2.90 (0.46) |  | 1.71 |
| Vocal Acoustics | 2.93 (0.57) | 3.17 (0.56) | 2.84 (0.54) |  | 2.63* |
| Gross Motor Expressivity | 1.95 (0.62) | 1.81 (0.55) | 2.00 (0.65) |  | -1.30 |
| Confident Expressivity | 4.01 (0.64) | 4.21 (0.52) | 3.94 (0.67) |  | 1.85 |
| Positivity of Speech Content | 3.86 (0.50) | 3.94 (0.53) | 3.83 (0.49) |  | 1.04 |

*Note.* A higher score indicates a greater degree or amount of behavior.

**p <* 0.05.
