# Supplementary material for: Emotional cues from expressive behavior of women and men with Parkinson’s disease
Source: PLoS One. 2018 Jul 2;13(7):e0199886. doi: 10.1371/journal.pone.0199886 (PMC6028092; doi:10.1371/journal.pone.0199886)
Supplement: S1 Supporting Information — (DOCX) [file pone.0199886.s004.docx]

**S1 Supporting information. Steps to develop expressive behavior domains.**

There were two steps to develop a series of summary scores for expressive behavior. First, we performed an unrotated principal component analysis to estimate the coherence of the entire set of 20 items as one overall expressive behavior component. The first unrotated principal component summarizes the interrelationship among all ICRP items. Armor’s index of internal consistency reliability, theta, which is computed on the eigenvalue of the first unrotated component, was found to be 0.86, similar to the computed Cronbach’s alpha of 0.83. Both internal consistency indices indicated high internal consistency among the 20 ICRP items. Consequently, we formed an Expressive Activation composite score by averaging all ICRP scores after first reverse-scoring the forward slouching and tremors items, which negatively loaded on the first component.

Second, we developed expressive behavior domains in order to provide more detailed information about behavioral aspects of expressive activation. We conducted a principal component analysis with varimax rotation using all ICRP items and found a six-component solution (see the below table). Six composite scores were created by averaging the scores of items that loaded on the same component. Internal consistency was assessed with Cronbach’s alpha. The first three components (Smile-Laugh, Conversational Engagement, and Vocal Acoustics) generally were lower-face, mouth-focused, and neurophysiologically related to fine motor control [1-3]. The Gross Motor Expressivity component focused on upper-face and axial movement, which is neruophysiologically related to gross motor control [2,3]. The loadings of the latter two components (Confident Expressivity, and Positivity of Speech Content) were conceptually coherent even though these two components exhibited lower internal consistency than the previous four components. For analytic completeness, we retained Confident Expressivity and Positivity of Speech Content but interpreted their results with caution. Confident Expressivity included behaviors which contribute to the expression of self-regulatory control.

The below table shows rotated component loadings for each behavioral item of ICRP in the six-component solution of the principal component analysis for people with Parkinson’s disease (*N* = 105^a^).

| ICRP item | Expressive behavior domain | | | | | |
| --- | --- | --- | --- | --- | --- | --- |
|  | Smile -Laugh | Conversational Engagement | Vocal Acoustics | Gross Motor Expressivity | Confident Expressivity | Positivity of Speech Content |
| cheek raising | 0.93 |  |  |  |  |  |
| lip corner puller | 0.88 |  |  |  |  |  |
| laughing | 0.81 |  |  |  |  |  |
| active facial expressivity | 0.74 |  |  |  |  |  |
| gesturing with arms |  | 0.74 |  |  |  |  |
| vocal speed |  | 0.68 |  |  |  |  |
| mouth closure during speech |  | 0.63 |  |  |  |  |
| talkativeness |  | 0.58 |  |  |  |  |
| blinking |  | 0.54 |  |  |  |  |
| articulation |  |  | 0.87 |  |  |  |
| loudness |  |  | 0.78 |  |  |  |
| vocal inflection |  |  | 0.73 |  |  |  |
| eyebrows raising |  |  |  | 0.72 |  |  |
| eyebrows pulling together |  |  |  | 0.72 |  |  |
| trunk and head movement |  |  |  | 0.66 |  |  |
| forward slouching |  |  |  | 0.45 |  |  |
| tremors |  |  |  |  | -0.86 |  |
| topic control |  |  |  |  | 0.53 |  |
| negative content |  |  |  |  |  | -0.80 |
| positive content |  |  |  |  |  | 0.60 |
| Eigenvalue | 3.22 | 2.91 | 2.51 | 2.16 | 1.57 | 1.28 |
| Explained variance (%) | 16.09 | 14.54 | 12.53 | 10.81 | 7.83 | 6.41 |
| Cronbach’s alpha | 0.90 | 0.74 | 0.80 | 0.60 | 0.38 | 0.19 |

*Note.* ICRP = the Interpersonal Communication Rating Protocol.

^a^In addition to 96 people, the extra nine people only having some missing data either in emotional indices or in expressive behavior items were also included in the principal component analysis in order to have a larger sample size.

**References**

1. Ekman P, Friesen W. Felt, false, and miserable smiles*.* J Nonverbal Behav. 1982;6: 238-252.

2. Rinn WE. The neuropsychology of facial expression: A review of the neurological and psychological mechanisms for producing facial expressions*.* Psychol Bull. 1984;95: 52-77.

3. Ross ED, Prodan CI, Monnot M. Human facial expressions are organized functionally across the upper-lower facial axis*.* Neuroscientist. 2007;13: 433-446.
